# Human multidien rhythms: Commentary for: “Multiday cycles of heart rate are associated with seizure likelihood: An observational cohort study”.

**DOI:** 10.1016/j.ebiom.2021.103698

**Published:** 2021-11-17

**Authors:** Timothée Proix, Maxime Baud

**Affiliations:** a Department of Basic Neurosciences, Faculty of Medicine, University of Geneva, Geneva, Switzerland; b Sleep-Wake-Epilepsy Center, NeuroTec and Center for Experimental Neurology, Department of Neurology, Inselspital Bern, University Hospital, University of Bern, Bern, Switzerland; c Wyss Center for Bio and Neuroengineering, Geneva, Switzerland

For centuries, scholars have sought temporal patterns in health and disease. Fertility, growth, sleep, mood, digestion, paroxysmal brain and cardiac disorders have all been proposed to be organized with temporal regularity. Ancient texts attest to the idea that celestial motion could cyclically influence the inner workings of the human body. Indeed, the circadian rhythm (∼24 hour) was found to be paced by the rotation of the earth and the ensuing day-night cycle but generated endogenously; a profound discovery that gained Jeffrey Hall, Michael Rosbash and Michael Young the 2017 Nobel prize in medicine and physiology.

What about human rhythms that are longer than circadian? Despite long-standing interest in the question, scientific understanding has remained very partial. Recently, key insights were gained from physiological recordings in ecological conditions over days, months or years. For instance, using continuous blood-pressure and heart rate monitors, the late Franz Halberg, a founder of chronobiology, proposed an about-weekly (so-called circaseptan) modulation of human physiology [1]. As a potential mechanism, about-weekly cycles of melatonin, aldosterone, growth hormone and cortisol were found in small human cohorts [2] as well as rhythmic changes in the metabolome and proteome in animal models [3]. In addition, co-existing about-monthly rhythms may not be limited to the menstrual period of women, as similar timing also exists in testicular functions and hormones [4]. Tim Bromage revealed faithful inner records of cycles longer than circadian: enamel deposition in teeth creates regular patterns at intervals of several days called Retzius striae (Figure 1) which, akin to dendrochronology, chronicle past metabolic cycles, characteristic of a given species [5] and individuals [6]. As these long rhythms are subject-specific, somewhat variable, and often composed of more than one period, the term multidien (multiday in 7) was coined across fields of investigation to encompass a range of shared periodicities among humans, primates and other mammals, including a continuum from weekly, bi-weekly to monthly and longer rhythms [3,6,8]. In the absence of readily-identifiable temporal cues, these free-running rhythms are putatively generated endogenously and may relate to basal metabolic rate [3].

**Figure 1:**
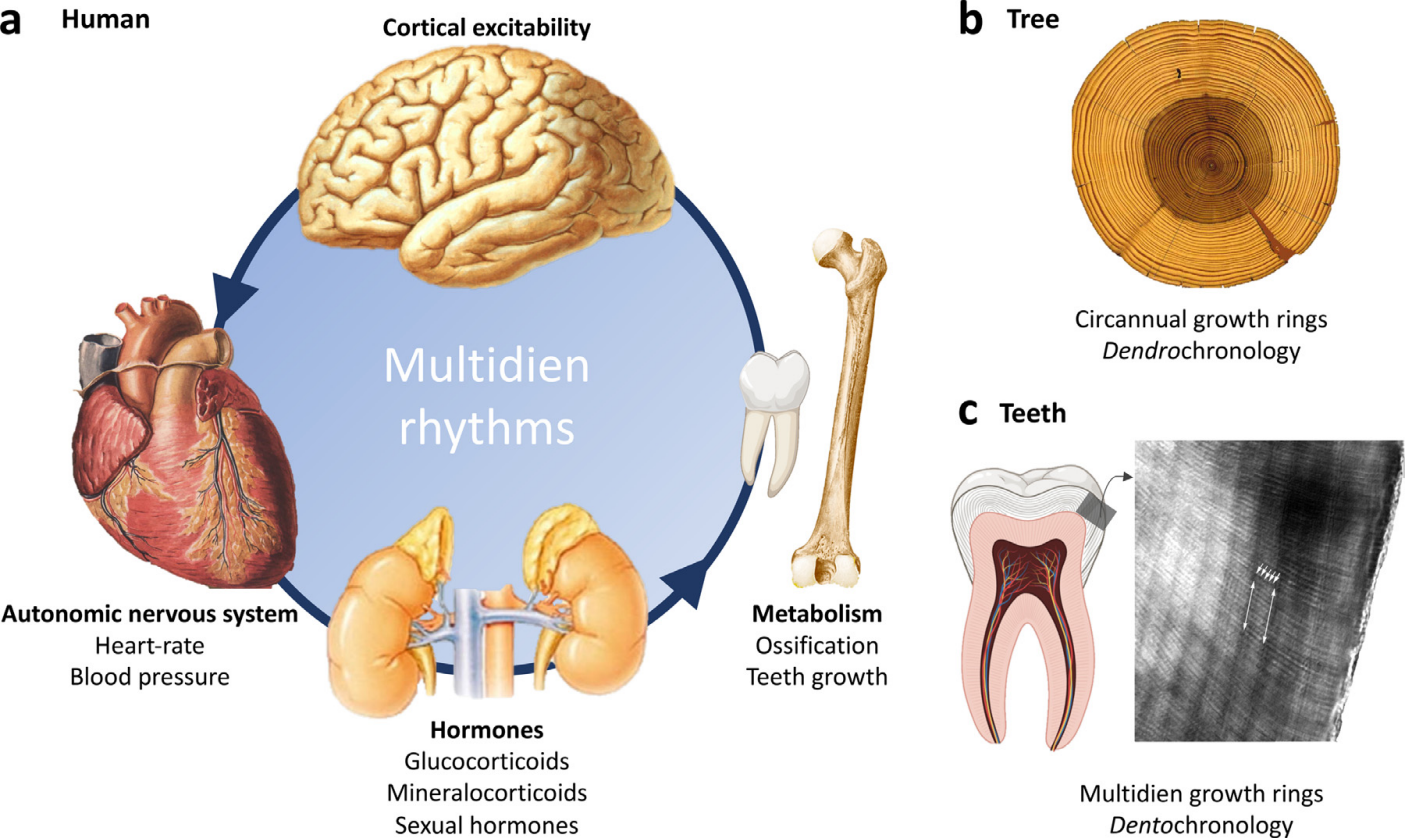
**Multidien cycles in mammals and circannual cycles in trees. a)** Known rhythms in different systems share periodicities over days. Classically, ovaries and sexual hormones in females undergo an about-monthly cycle, but testis, cortical excitability, heart rate and blood pressure also all fluctuate with about-weekly to about-monthly periods. Secretion of steroid hormones from the adrenal glands, excretion of sodium by the kidneys and hard-tissue growth undergo an about-weekly cycle. **b)** An established method to date the age of timber or trees is called dendrochronology. Here, each ring results from one full revolution of the earth around the sun. **c)** A similar strategy of counting rings can be used to date human teeth for anthropological purposes or to determine the period-length of multidien (long arrows) and circadian (short arrows) cycles specific to species or individuals. Each darker ring correspond to one full multidien cycle. Original figure partly created with biorender.com. Micrograph taken from (3).

In the field of epilepsy, with the development of implantable EEG systems and advances in data processing techniques, tracking of neural activity over long periods has recently become possible, leading to the (re-)discovery of multidien rhythms of epileptic brain activity that underlie the historically-observed periodicity in seizure timing [8], suggesting a connection to systemic rhythms.

In the October, 2021 issue of EBioMedicine, Karoly et al. report on a prospective study associated with the “My Seizure Gauge” challenge sponsored by the American Epilepsy Foundation, in which both heart rate and seizures were tracked longitudinally and non-invasively over months, with the simple use of smartwatches and seizure diaries [7]. They reveal an exciting and promising finding: the existence of multidien rhythms of heart rate in the majority of people with epilepsy (here 29 from 31) and healthy controls (here 12 from 15). They further show that about half of participants with epilepsy displayed significant correlations between their heart rate cycles and reported seizures. The results strikingly resonate with prior work done on seizure cycles based on more invasive chronic EEG recordings, where the same seizure periodicities were found across a majority of people with epilepsy [9].

Methods to characterize very long physiological time series is an area of ongoing analytical refinement. Subtle issues come into consideration when seeking periodicity and synchrony in non-stationary biological signals [10]. This study has methodological strengths in that regard. First, long recordings that capture several cycles (here ≥ 4) and events (here ≥ 20 seizures) in each individual provide confidence in the results. Second, analyses based on the well-established wavelet transform rightfully accommodate for the natural variability of free-running multidien cycles [10] and matched original methodology used for the discovery of multidien rhythms of epileptic brain activity [8]. Finally, the use of a non-invasive recording method of peripheral physiology is practical and advantageous over implantable EEG devices. However, the lack of inclusion of EEG recordings - the gold-standard method for objectively documenting seizures - is a clear limitation of the study acknowledged by the authors: it precludes a direct comparison between brain and heart multidien rhythms. Future studies including parallel multimodal physiological monitoring will undoubtedly deepen our appreciation of synchrony or independence among different multidien rhythms.

Yet, as it stands, the study already challenges our fundamental understanding of biological rhythms. While synchrony between two circadian rhythms is trivial (they are both paced by a master clock in the supra-chiasmatic nucleus), the prime question of the exact way multidien heart rate, seizure and other cycles influence each other remains open, as no core multidien clock has yet been identified. Synchronization can either be set by a single (and so far unknown) common factor that would pace their rhythms, or by direct coupling relationships, in a framework of circular causality. The latter can be described in dynamical terms as a set of coupled oscillators and could lead to interesting testable predictions such as the effect of phase shift of one rhythm on the others. Understanding these interactions will necessitate a multidisciplinary approach, leveraging multimodal recordings over long duration, dynamical system modelling, but also investigations of the basic molecular mechanisms of slow rhythms, might they involve metabolic processes, hormones, and/or the autonomic nervous system. Undoubtedly, future studies will uncover a large number of other physiological variables that vary rhythmically over long-time scales, be they multidien or even circannual.

This study also has consequences for present and future clinical practice. It confirms the importance of measuring vital signs at several time instants to fully assess the cardiac physiology of any given person. It also places the epileptic brain back into a system of internal rhythms, abating boundaries between medical fields. For people with epilepsy for whom heart rate cycles correlate with seizure occurrence, straightforward heart rate measurements will help probe the individual propensity to have seizures at certain times, marking a crucial advance towards the Grail of seizure forecasting. In summary, the discovery of multidien rhythms in human physiology will have a profound effect on the preventative medicine of tomorrow through novel abilities to forecast recurrent paroxysms and mitigate their risk with use of chronotherapy.

## Contributors

TP and MOB contributed equally to this commentary.

## Declaration of Competing Interest

The authors declare no conflict of interest related to this work.
